# Surface and Size Effects in Spin-Crossover Nanocrystals

**DOI:** 10.1186/s11671-017-1844-z

**Published:** 2017-02-08

**Authors:** Iurii Gudyma, Victor Ivashko, Andrej Bobák

**Affiliations:** 1 Department of General Physics, Yuriy Fedkovych Chernivtsi National University, Kotsjubynskyi Str. 2, Chernivtsi, 58012 Ukraine; 2 Department of Theoretical Physics and Astrophysics, Faculty of Science, P.J. Šafárik University, Park Angelinum 9, Košice, 04154 Slovak Republic

**Keywords:** Spin-crossover nanoparticles, Ising-like model, Surface, Size, Cooperative effects

## Abstract

We perform Monte Carlo simulations to analyze the surface and size effects in spin-crossover nanocrystals using an Ising-like model including surface and core intermolecular interactions. The consequences of downsizing effect on the transition temperature and the width of hysteresis as finger of the system cooperativity are discussed. The critical temperature is calculated using the real-space renormalization method. The obtained results are in agreement with the experimental data.

## Background

The spin-crossover molecular magnets are a new class of coordination inorganic complexes with *d* ^4^−*d* ^7^ electronic configuration of metal ion orbitals, situated in the centre of the organic octahedral ligand field [1–3]. Depending on the specific geometry and the strength of the ligand field, such materials possess two stable states: the one with low-spin (LS) configuration with diamagnetic properties and another one with high-spin (HS) configuration showing paramagnetic behavior. A variation of temperature, pressure, or light irradiation may induce transition from LS state to HS one. We focus on iron(II) compounds with coordination number 6, due to their practical applicability as sensors, data storage, switching, display, and visualization systems. The attractiveness of practical applications of these materials lies in the possibility to design IT devices with nanosized unit cells of about 4 nm ^3^ [4]. For these compounds, the total spin number for LS and HS configurations is respectively *S*=0 and *S*=2 [1].

The recent synthesis of spin-crossover materials at the nanoscale offers interesting prospects [5, 6]. Decreasing the size of the molecular system from macroscopic (bulk) down to nanometric scale can provide unique insight into fundamentals of spin transition properties, with applications to nanoelectronics and spintronics [7, 8]. The group of Bousseksou [9] is particularly advanced in the synthetic elaboration of nanometric thin films and nano-sized patterns that are obtained by electron beam lithography and in the application of an external perturbations in the hysteresis loop of spin-crossover materials leading to an irreversible switching of their physical properties. The group of Létard [10] successfully synthesizes the spin-crossover nanoparticles prepared by the reverse micelle technique, which exhibit thermal hysteresis at room temperature. Unconventional soft-lithographic techniques have also successfully been applied to pattern spin-crossover nanoparticles and to perform reliable nanopatterns of crystals and molecular deposits [11]. There are exists a lot of reports [12–16] which deal with the problems of formation and study of nanostructures which are basing on the spin-crossover materials. In the case of spin-crossover complex, the most important question is how downsizing effect influences the transition temperature and the width of hysteresis loop as finger of the cooperativity.

From experiments [5, 17], it is known that upon a reduction of size, the spin transition becomes incomplete with residual high and low spin fraction. Also, it is observed that the transition temperature is shifted downwards and the hysteresis loop is narrowing and almost vanishes for smaller particles. For nanosize particles, a relatively gradual thermal spin crossover often occurs without clear existence of thermal hysteresis, although the equilibrium temperature of the transition is almost unaffected by the decrease of the size [18]. The evolution of the spin-crossover properties is not only dependent on the size but also depends critically on the chemical nature of the compounds and is mostly determined by the structural arrangement of ligands. The main experimental observations on the size reduction effects in spin-crossover compounds are overviewed in paper [19]. The experimental studies have confirmed the role of the surface effects which lead to unusual and non-trivial size dependence of their bistable character.

In the present study, we have carried out a systematic Monte Carlo simulations basing on Ising-like model of small spin-crossover system with a view of agreement with experimental data. The brief theoretical descriptions of proposed methods are given in the next section. The obtained results of numerical simulations are described in the “Results and discussion” section. In the last section, the summarization of the results is made.

## Methods

Phenomenologically, the interactions in molecular spin-crossover nanoparticles can be modeled in a simplest way by the Ising-like Hamiltonian

1 $$\begin{array}{@{}rcl@{}} &H = -h \sum_{i} s_{i} - \sum_{<ij>}^{c} J_{ij}^{c} s_{i}s_{j}& \\ & - \sum_{<ij>}^{s} J_{ij}^{s} s_{i}s_{j}- \sum_{<ij>}^{c-s} J_{ij}^{c-s} s_{i}s_{j}.& \end{array} $$

Here, *s*_*i*_ is a fictitious spin (pseudospin) operator which has two eigenvalues ±1, corresponding to the HS and LS states of respective *i*-s molecule, <*i* *j*> denotes the summation over all the nearest-neighboring spin pairs. The Ising-like Hamiltonian describes the elastic interaction between spin states via the near-neighbor coupling of two-level units. The intersites short-range coupling constants $J_{ij}^{\alpha }$ are parameters of the theory, where *α*=*c*,*s*,*c*−*s* correspond to occupied pairs of core sites, surface sites, and core-surface sites, respectively.

A simple phenomenological approach to the problem is the mean field approximation with the nearest-neighbor interactions in the form $\sum _{j (j \not = i)} J_{ij}^{\alpha }\approx zJ^{\alpha }$ with coordination number *z* (the number of first neighbors of a given molecule in the lattice). We assume that *J* ^*α*^ are positive (ferromagnetic-type interaction) and satisfy the following inequalities: *J* ^*c*^≥*J* ^*c*−*s*^≥*J* ^*s*^. For simplicity, we will hereinafter consider the case whit *J* ^*c*^=*J* ^*c*−*s*^. Thus, the thermal hysteresis width should be studied depending on the ratio of surface interaction and core interaction *η*=*J* ^*s*^/*J* ^*c*^ in nanoparticles, as well as surface-to-volume ratio *S* *A*:*V*, which in this case, the ratio of number of surface sites *N*_*s*_ to the number of sites in the core *N*_*c*_ of nanoparticles.

The effective alternating field which describes the resulting environmental action on single molecular magnet is

2 $$ h=-(\Delta -k_{B}T \ln g).   $$

where *Δ* stands for the energy difference between HS and LS states (it is the enthalpy change associated with the LS→HS conversion) for an individual spin-crossover molecule, *g*=*g*_HS_/*g*_LS_ is the degeneracy ratio between HS and LS energy levels, *k*_*B*_ is the Boltzmann’s constant. It is considered that the value *Δ* is fully formed by the ligand environment of the transition metal ion, this is why it is called the ligand field. Thus, in noninteracting system, the critical temperature $T_{c}^{0}$ at which mole fractions of the LS and HS states are equal corresponds to a zero effect field and, as a result, one obtains $T_{c}^{0}=\Delta /(k_{B}T \ln g)$. The Hamiltonian (1) describes in a convenient way the behavior of spin-crossover system, where the control parameter is a temperature *T*. If the critical temperature in purely Ising model is smaller than the critical temperature in noninteracting model, then the gradual spin transition from LS to HS takes place by increasing the temperature. Otherwise, the spin transition is discontinuous and is associated to a first-order phase transition.

In this work, the spin-crossover system with free boundary conditions described by the Hamiltonian (1) has been examined by Monte Carlo (MC) simulations employing the standard Metropolis algorithm [20–22]. The MC simulations were performed for the 3D Ising-like system on the cubic lattices of various sizes and 1000 MC steps which are sufficient to realize a steady state. Respectively, the number of the nearest neighbors *z* is equal to six. The main steps of Metropolis algorithm are (i) to fix the temperature, (ii) to fix initial spin configuration, (iii) to find the system energy for initial configuration, (iv) to flip arbitrarily one spin from the system, (v) to find the system energy of new configuration, and (vi) to evaluate the transition probability of new configuration. The probability of system transition from an initial configuration with energy *E*_*i*_ to a final configuration with energy *E*_*f*_ is given by exp[−*β*(*E*_*f*_−*E*_*i*_)]. Depending on the value of transition probability, this configuration may be accepted or rejected. If transition is accepted, the fictitious magnetization *m* of this configuration is found. This is one MC step. To have a good view of evolution of system order parameter, it is needed to carry out the numerical simulation for a sufficient number of MC steps, for which the system reaches the stationary regime. For each temperature, the resulted system magnetization *m* was found as the average on the last MC steps *N*_MC_ from stationary regime of trajectory

3 $$ m=\frac{1}{N_{\text{MC}}} \left|\left< s \right>\right|,   $$

where the average of fictitious spin has the following form

4 $$ \left< s \right> =\frac{1}{L^{3}}\sum_{i} s_{i}.   $$

Here, *L* is lattice size of 3D cubic Ising model or the number of molecules on the edge of the cube.

The examined system was initialized with all spins down and temperature low enough to maintain such spin configuration. If the temperature is increasing, the spins are randomly flipped, and for a certain temperature value, they become ordered in opposite state. The relation between the magnetization of Ising model and natural order parameter of studied spin-crossover system, which is the fraction of HS molecules *n*_HS_, is the following

5 $$ n_{\text{HS}}=\frac{m+1}{2}.   $$

In point of fact, the high spin fraction *n*_HS_ is the mean number of molecules in the HS state.

The behavior of spin-crossover system of various size and surface-to-volume ratio of sites for different values of ratio between surface and core intermolecular interactions was examined basing on the thermal transition curves obtained from Monte Carlo simulations of Hamiltonian (1). For the following Monte Carlo simulations, the simple cubic lattice consisting of *L* ^3^ lattice points was used. The obtained numerical results based on these approaches are presented in the next section.

## Results and discussion

**Fig. 1:**
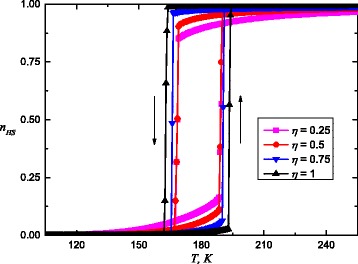
The spin transition curves calculated by Monte Carlo technique for fixed values of ratio between surface and core interactions *η*. The system’s parameters are the following: *g*=150, *Δ*=900 *K*, *J* ^*c*^=85 *K*

At the beginning, the simulation was performed on the thermal transition curve for fixed values of ratio between surface and core interactions as can be seen in Fig. 1. For numerical calculations hereinafter, we chose the following set of system parameters: *g*=150, *Δ*=900*K*, *J* ^*c*^=85*K*, the coordination number *z*=6 and 1000 Monte Carlo steps per Kelvin degree. In this particular example, we used four significant values of ratio between surface and core interactions that focus our attention on the typical dependence of transition curves with decreasing a surface interaction between transition metal ions. One can note that a first-order phase transition accompanied by a thermal hysteresis can take place. The curves are calculated for a finite 3D system (12 × 12 × 12 molecules with free boundary conditions). Naturally, the ranges of overlapping of two hysteretic branches depend on the system size but we will return to this issue below.

From this plot, we can analyze the changes of the hysteresis between cooling and heating processes with decreasing of the parameter *η* which reflect the ratio of surface interaction and core interaction. There is a collapse of the hysteresis inasmuch as its area is reduced not only by narrowing the loop but also due to failure of the saturation. The hysteresis loop vanishes showing a progressive loss of cooperativity, and this picture agrees with the experimental observations.

**Fig. 2:**
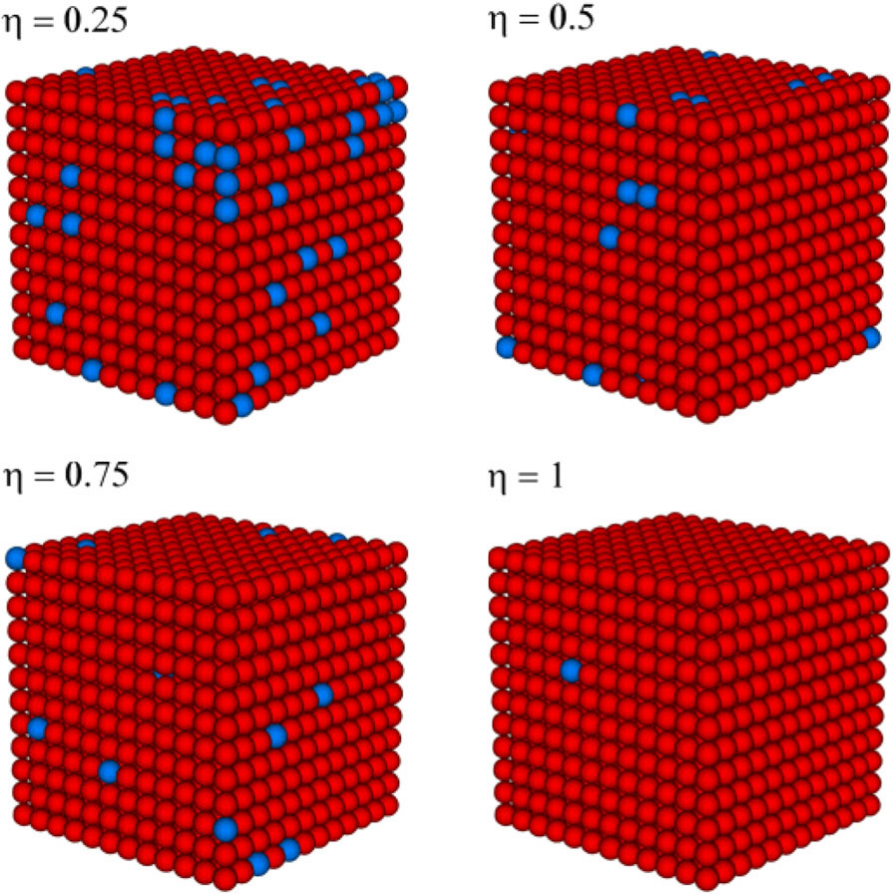
The snapshots of 3D system for various values of *η* at constant temperature *T* = 255 K

Three-dimensional snapshots at various *η* are presented in Fig. 2 for the same system parameters. Calculations are made at high temperature *T*=255 *K* exceeding the existence region of hysteresis. Here, the red balls indicate molecules in HS state, and the blue balls represent the molecules in LS state. Although as it is shown in Fig. 2, the phase transition on the nanoparticle’s surface is not completed. The smaller is the value of the ratio *η*, the more nodes on the nanoparticle surface do not participate in the phase transition. This result is easy to understand, if we return to Fig. 1. The reduction of surface interaction not only leads to a decrease in residual magnetization but also to the saturation of hysteresis loop value. In fact, a decrease in surface interactions prevents saturation of the hysteresis curve.

Closer look at Figs. 1 and 2 leads to the conclusions about an incomplete phase transition for small *η*. Note that in the experiment, an incomplete phase transition can look like the lack thereof. It is remarkable that the solid solution of “infinite size” containing only 20% of iron exhibits a complete spin transition between the pure low-spin state at low temperatures and the pure high-spin state at high temperatures [23].

**Fig. 3:**
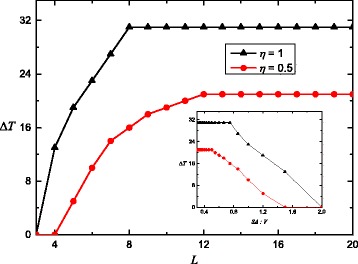
Size dependence of the width of the thermal hysteresis *Δ* *T*. *L* is the number of molecules on the edge of the cube

Further calculations have been performed for the changes of hysteresis width as a function of the size *L* of the system with free boundary conditions. In order to characterize the changes of hysteresis loop width for different size, we have to derive the transition temperatures during cooling and heating process *T*_down_ and *T*_up_, respectively. These temperatures are defined as equilibrium temperatures at which the fraction of HS molecules takes the value *n*_HS_=0.5. The width of the thermal hysteresis *Δ* *T* is calculated by $\Delta T=T_{\text {up}}^{eq}-T_{\text {down}}^{eq}$. The obtained results are shown in Fig. 3. Simulations were performed on cubic lattices of size *L*×*L*×*L* (see Fig. 2) for a sequence of finite sizes up to *L*=20. They show a general tendency that the hysteresis cannot take place for particularly small nanoparticles. In excellent agreement with experiment reported in [24], we have found that the hysteresis width vanishes at a critical particle size. This is a consequence of the different intrinsic cooperative nature of the physical phenomenon on surface and inside the solid.

Here, the red circles indicate the values of hysteresis width for *η*=0.5, and the black circles correspond to hysteresis width for *η*=1.0. According to Fig. 3, the hysteresis width increases with the system size increasing up to the saturation. The weakening of the intermolecular interaction on the surface prevents the reaching of fast saturation. Similar analyzes were performed for the other choices of parameter *η*=0.5 which is investigated in the present work. Figures 1, 2 and 3 indicate that the magnitude of size effects is directly related to the difference between the intermolecular interactions in the volume and on the surface. This is due to the fact that sufficient cooperative interactions between the molecules are precondition for the observation of thermal hysteresis loops during the spin transition.

As the sizes decrease, the increase in surface area to volume ratio makes the surface effect more pronounced. At the nanoscale level, the surface effect is ambiguous because it can act in either a detrimental or beneficial role depending on the application. If the dimension of the overall system is given *L*, then the volume of ideal cubic nanoparticle is

6 $$ V=L \times L \times L   $$

and the surface area of nanoparticle is

7 $$ A=6 \times L \times L.   $$

In this simplest case, the surface to volume ratio is then given by

8 $$ \text{SA}:V=6 / L.   $$

It is clear that magnitude *Δ* *T* is dependent on the surface-to-volume ratio SA:*V* as an inversely proportional quantity. This is illustrated by the inset for Fig. 3. Thereby, the size is the main factor governing the spin transition behavior.

**Fig. 4:**
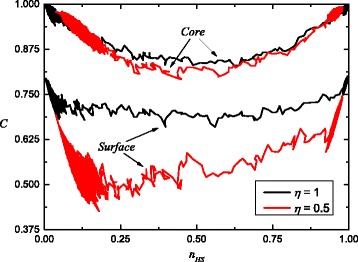
Correlation factors *C* for core and surface components

The transition is abrupt and the molecules in the HS state are forming clusters if we move along the heating curve. The process of clustering can be estimated using the correlation factor

9 $$ C = \frac{1}{zN}\sum_{i=1}^{N}\left| s_{i} \sum_{j=1}^{z} s_{j}\right|,   $$

where *N* is the total number of molecules in a nanoparticle. The second sum is over the nearest neighbors of *i* site. It is noteworthy that this factor is maximal at the beginning and at the end of transition, when all spins are parallelly aligned, and is minimal during the transition, in the neighborhood of the point when the numbers of high spin and low spin molecules are equal. The value of this minimum depends on interactions: it decreases with lower interactions and can become practically zero in the absence of interactions. We show in Fig. 4 the correlation factor corresponding to first-order neighbors for core and surface components. As can be seen, both core and surface molecules have a strong correlation dependence. But even for *J* ^*s*^=85*K*, surface molecules do not reach saturation due to free boundary condition. We remind that the word “cluster” refers to a group of spin whose values are correlated with one another [25]. If the cluster in the core may cover the entire volume, then on the surface, it does not occur at the investigated conditions. This result corresponds to the snapshots shown in Fig. 2.

**Fig. 5:**
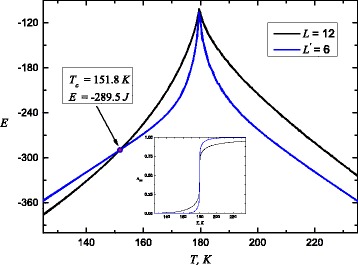
Computer simulation of internal energy per spin *E* versus temperature *T* at fixed intersites couplings *J* ^*c*^=*J* ^*s*^=44*K*. *Inset*: the appropriate spin transition curves

As a last point of this paper, we consider second-order transitions. Figure 5 shows the internal energy per spin *E* as a function of temperature *T*. From this plot, it is possible to locate the critical point. One may plot *E* versus *T* for pair of size *L* and *L* ^′^ and estimate *T*_*c*_ from common intersection point of these curves. *L* ^′^ is a new system size obtained after system dividing into blocks with size 2^*d*^, where *d* is a dimension of system, therefore *L* ^′^=*L*/2. The spins on the lattice are grouped into blocks and form a coarser lattice. This is a base of the real-space renormalization method. Original and rescaled systems have the same correlation length and hence the same critical temperature.

The work is a natural extension of previous studies in this field [22, 26].

## Conclusions

We have presented an analysis of an Ising-like model with small number of sites and different intermolecular interaction on the surface and in the core of a quasiparticle that describes the spin-crossover nanocrystals. From Monte Carlo simulations for 3D configuration basing on Metropolis transition probabilities, the thermal transition curves were obtained. For spin-crossover nanosystems, the narrowing of hysteresis width by decreasing of the intermolecular interaction on surface is detected. It is found that in this case, the phase transition can be only incomplete. The appearance of HS and LS residual fractions which are observed for the majority of spin crossover complexes can be explained in the framework of the proposed approach.

The width of the temperature hysteresis, which is unchangeable for large system size, is narrowing by reducing the number of magnetic molecules in the nanoparticle. The hysteresis width vanishes at a critical particle size. The size and the surface properties are the main factors governing the spin transition behavior. We also show a difference in the correlation effects on the surface and in the core of the nanoparticles. A Monte Carlo renormalization group calculation of the critical temperature of the three-dimensional system is presented.

The obtained results of numerical calculations are in agreement with the experiments [1, 2, 27]. It is worthwhile to note that the observed correlation between phase transition in spin-crossover materials (critical temperature and hysteresis width) and sizes of the bulk crystals/nanoparticles is uncommon through experimental difficulties.
